# Integrating BERT Embeddings and BiLSTM for Emotion Analysis of Dialogue

**DOI:** 10.1155/2023/6618452

**Published:** 2023-05-29

**Authors:** Zhinan Gou, Yan Li

**Affiliations:** ^1^School of Information Technology, Hebei University of Economics and Business, Shijiazhuang, Hebei 050061, China; ^2^School of Information Engineering, Shijiazhuang Vocational College of Finance & Economics, Shijiazhuang, Hebei, China

## Abstract

Dialogue system is an important application of natural language processing in human-computer interaction. Emotion analysis of dialogue aims to classify the emotion of each utterance in dialogue, which is crucially important to dialogue system. In dialogue system, emotion analysis is helpful to the semantic understanding and response generation and is great significance to the practical application of customer service quality inspection, intelligent customer service system, chatbots, and so on. However, it is challenging to solve the problems of short text, synonyms, neologisms, and reversed word order for emotion analysis in dialogue. In this paper, we analyze that the feature modeling of different dimensions of dialogue utterances is helpful to achieve more accurate sentiment analysis. Based on this, we propose the BERT (bidirectional encoder representation from transformers) model that is used to generate word-level and sentence-level vectors, and then, word-level vectors are combined with BiLSTM (bidirectional long short-term memory) that can better capture bidirectional semantic dependencies, and word-level and sentence-level vectors are connected and inputted to linear layer to determine emotions in dialogue. The experimental results on two real dialogue datasets show that the proposed method significantly outperforms the baselines.

## 1. Introduction

With the continuous progress of science and technology, more and more consumer groups use intelligent products. In order to improve the general performance of intelligent products and user experience, human-computer interaction has become the top priority of intelligent products related research. Dialogue system is an important application of natural language processing in human-computer interaction, for example, both task-oriented dialogue system and open-domain dialogue system, such as intelligent customer service and chatbots. In addition to generating the appropriate response, they also focus on the subjective feelings of users for giving users a good experience in the conversations [1].

Emotion analysis of dialogue system aims to classify the emotion of each utterance that refers to the attitudes, opinions, and emotional tendencies in the dialogue process [2]. Emotion analysis is crucially important to dialogue system and contributes to the good experience of user, i.e., when the user speaks in intelligent customer service system, “I bought it last week, and it's broken,” user describes objective facts about product defects and expresses his dissatisfaction with the product by using an angry mood. It follows then that emotion analysis of dialogue is great significance to the practical application of customer service quality inspection, intelligent customer service system, chatterbots, and so on.

The emotion classification methods include dictionary-based models, machine learning models, and deep learning models. Dictionary-based models use the polarity and intensity value of emotion terms, the intensity value of degree terms, and the value of negative terms to classify the emotion of sentences. However, dictionary-based models depend on emotions dictionary that is labor-intensive and time-consuming. In addition, multiple emotions dictionary is difficult to build. Each of machine learning models has advantages and disadvantages in certain situations. In machine learning methods, a sentence emotion classifier can be trained by inputting a large number of sentences with emotional labels and predict the emotion of new sentences. Machine learning methods mainly include k-nearest neighbor, Naive Bayes [3], decision tree, and support vector machine [4], which are extensively used in emotion classification. However, machine learning methods require the construction of feature model, which is inefficient and time-consuming.

The existing deep learning models of emotion analysis mainly use word vector and are based on recurrent neural network. Deep learning-based architecture is superior to the machine learning methods through accuracy and low complexity [5]. However, neural network models only input word-level vectors into neural network and predict the emotion. Sentence-level vectors are not considered and explored to neural network models. As a result, local information and global information are not completely accurate. In addition, although emotion analysis of sentences in a dialogue system is very important, there is no dialogue emotion analysis based on BERT embedding and BiLSTM.

Based on existing research, for emotion analysis, the proposed combination of the BERT (bidirectional encoder representation from transformers) model and the BiLSTM (bidirectional long short-term memory) model is used to model global and local information. The major contributions of the paper are summarized as follows:

- The architecture based on BERT embeddings and BiLSTM is proposed and constructed to determine emotions from dialogue.
- As a feature selection model, BERT extracts word-level and sentence-level vectors from the inputting data and is embed into the neural network architecture. And then, word-level vectors are combined with BiLSTM to concatenate sentence-level vectors for emotion analysis.
- To evaluate the proposed emotion classification model, experiments are conducted on two real dialogue datasets. The experimental results show that the proposed method significantly outperforms the baselines.

To the best of our knowledge, this is the first method of integrating BERT embeddings and BiLSTM for emotion analysis of dialogue. This paper goes as follows. The related works are introduced in the second part. The third part details the emotion classification model based on BERT embeddings and BiLSTM. Next, the experimental is described, and the results are analyzed by comparing with baselines. The summary of the research is in the last section.

## 2. Related Works

In this section, we review some related works on feature vectorization and LSTM in detail. Based on analyzing the limitations of related works, we present a method of integrating BERT embeddings and BiLSTM for emotion analysis of dialogue to address these limitations.

### 2.1. Feature Vectorization

The vectorization of text features is a key part of the classification tasks. In general, words are mapped into a unified vectors space. One-hot representation is the simple method of feature vectorization. However, one-hot representation is traditional rule-based or statistics-based natural semantic processing methods that only treat a word as an atomic symbol neglecting the semantic relationship of words. In addition, one-hot representation leads to feature vector with high dimensions that increases the computational complexity and affects the subsequent classification. As distributed representation, latent semantic analysis [6], probabilistic latent semantic analysis [7], and latent Dirichlet allocation [8] can extract features for text similarity calculation [9] and text classification [10], and these models also neglect the semantic relationship of words [11]. Word embedding contains more information and maps a word into a distributed representation. Each dimension in word embedding vectors space has a specific meaning. There are many models to generate word embedding. Word2Vec is a lightweight neural network that only includes input layer, hidden layer, and output layer. The Word2Vec framework mainly includes CBOW and skip-gram models according to the difference of input and output [12]. Skip-gram acquires the semantic vector of a word according to text context, and CBOW learns the text context probability of term.

Bidirectional encoder representations from transformers (BERT) is a self-coding pretrained model of language representation [13]. There are different types of tasks that are used to design BERT. The first task randomly selects some words that are replaced with a special symbol (MASK), and then, the model learns to fill in these places according to the labels given. The second task adds a sentence-level prediction that predicts whether two sentences are continuous for learning the relationship between the continuous segments of text. Some researches of various applications based on BERT to vector text features are proposed [14–16]. In sentiment classification, a Chinese sentiment classification model based on pretrained BERT is used to extract the text abstract features of a single Chinese character based on the context semantic relationship [17]. A long-text classification method of Chinese news uses BERT pretrained language model to complete the sentence-level feature vector representation of a news text and captures global features by using the attention mechanism to identify correlated words in text [18]. A novel BERT-based framework is proposed to show the enhanced performance obtainable by combining latent topics with contextual BERT embeddings [19]. A framework based on BERT and CNN with attention mechanism is used to sentiment classification of microblog [2]. In the financial field, BERT and CNN are combined for the classification of candidate causal sentences [20]. In summary, BERT has excellent performance and is widely used in various fields of text classification.

### 2.2. LSTM

Long short-term memory networks (LSTM) is a special RNN network, which is designed to solve the long dependency problem [21]. Both traditional RNN and LSTM transmit information from front to back, which has limitations in many tasks. Such as POS tagging tasks, the POS of a word is related not only to the word before but also to the word after. In order to solve this problem, bidirectional long short-term memory (BiLSTM) is proposed and composed of two LSTM networks. The idea is to connect the same input sequence to the forward LSTM and backward LSTM, respectively, and then connect the hidden layers of the two LSTM networks together to the output layer for prediction. BiLSTM already has a variety of applications in technology. BiLSTM-based systems can learn to translate languages [22], document summaries [23], speech recognition [24], dialogue system [25], predicting disease [26], and so on. In sentiment classification, the BiLSTM, BiGRU, and CNN model are integrated and proposed for sentiment classification [5]. A hybrid model of sentiment classification is proposed, which is based on BERT, BiLSTM, and a text convolution neural network [27]. In the legal area, a shallow network with one BiLSTM layer and one attention layer is used to perform Portuguese legal text classification [28]. ABLG-CNN is text classification model, which is attention-based BiLSTM fused CNN with gating mechanism for Chinese long-text classification. In this model, the attention mechanism is used to calculate context vector of words to derive keyword information. BiLSTM captures context features, and CNN captures topic salient features [29].

However, the existing research of emotion analysis has not use BERT word-level embeddings and sentence-level embeddings for extracting local information and global information from text. In addition, although emotion analysis of sentences in a dialogue system is very important, there is no dialogue emotion analysis based on BERT embedding and BiLSTM. In this study, the architecture based on BERT embeddings and BiLSTM is proposed and constructed to analyze emotions from dialogue. Details of the proposed architecture are described in methods section.

## 3. Methods

### 3.1. Research Framework

**Figure 1:**
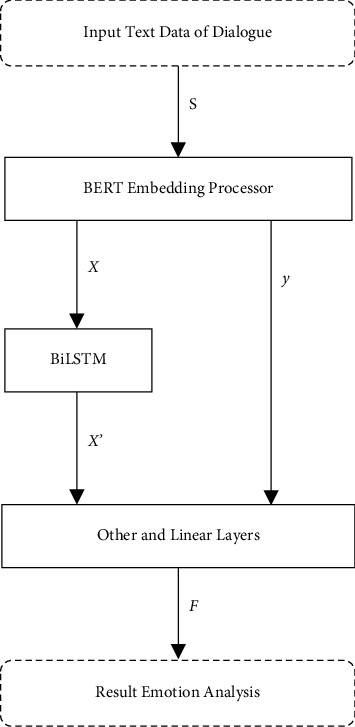
Framework of emotion analysis model.

Figure 1 shows the research framework of emotion classification of dialogue based on the BERT embeddings-BiLSTM model. First, put the data of dialogue into a BERT embedding processor to generate word-level and sentence-level vectors. Then, word-level feature is processed by BiLSTM. Finally, the processed word-level vectors and sentence-level vectors are connected and inputted to linear layer for emotion analysis of dialogue.

### 3.2. BERT Embedding Processor

This paper selects BERT that has better feature representation ability. In BERT embedding processor, the text of dialogue is converted into word vectors and sentence vector, respectively. Sentence vector represents the semantics feature of sentences and uses the output of the penultimate layer. Sentence vector is a pool output that is a special classification token (CLS). The final hidden state corresponding to CLS token is used as the aggregate sequence representation for classification tasks. Word vector is a sequence output, which corresponds to the last hidden output of all the words in the sequence. Sentence-level features can represent the original semantics of the whole sentence without reprocessing. Processed by BiLSTM, the dependency relationships between words in word features can be mined, which is a good supplement to sentence feature modeling.

In this section, the sentences of dialogue S are input to BERT embedding processor, and then, word vectors *X* = {*x*_1_, *x*_2_,…, *x*_*m*_} and sentence vectors *y* are generated, where *m* is the maximum sequence length of sentence. Word vectors *X* are sent to BiLSTM for further processing.

### 3.3. BiLSTM

On the basis of word vectors *X*, *X*′ is the long-distance dependence of the word vector of the dialogue sentence that is further learned by the BiLSTM model. The processed feature *X*′ is combined with the sentence-level feature *y* of the sentence, which can better represent the feature of the dialogue sentence. The BiLSTM neural network structure model is divided into two independent LSTMs. The input sequences are input into two LSTM neural networks in positive and reverse order, respectively, for feature processing. The splicing of the two output vectors is the processing word vectors *X*′ that is used as the final feature expression of the words of sentence.

**Figure 2:**
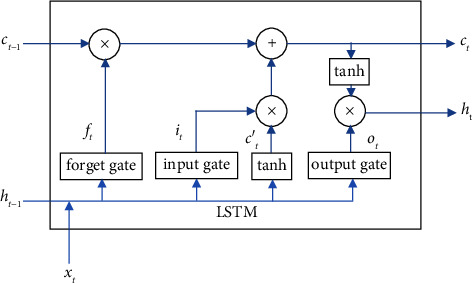
The LSTM neural network model.

LSTM consists of three gates: forget gate, input gate, and output gate. The forget gate controls the information obtained from the previous unit and determines the information discarded. The input gate controls the proportion of the input information added to the unit state. The output gate controls the update of the current memory state and the output of the hidden layer. The LSTM neural network is shown in Figure 2.

The text continues here. In LSTM neural network model, forget gate is used to choose the historical information retained by the cell state, input gate controls the proportion of inputting new information saved to the cell state, and output gate determines the final output information. The mathematical modeling of LSTM unit at the state of the *t*-th position is shown in the following equations:

(1) $$\begin{array}{c} f_{t}=\sigma \left(W_{f}\left[h_{t-1},x_{t}\right]+b_{f}\right)\text{,} \end{array}$$

(2) $$\begin{array}{c} i_{t}=\sigma \left(W_{i}\left[h_{t-1},x_{t}\right]+b_{i}\right)\text{,} \end{array}$$

(3) $$\begin{array}{c} c_{t}^{\prime }=\tan h\left(W_{c}\left[h_{t-1},x_{t}\right]+b_{c}\right)\text{,} \end{array}$$

(4) $$\begin{array}{c} c_{t}=f_{t}*c_{t-1}+i_{t}*c_{t}^{\prime }\text{,} \end{array}$$

(5) $$\begin{array}{c} o_{t}=\sigma \left(W_{o}\left[h_{t-1},x_{t}\right]+b_{o}\right)\text{,} \end{array}$$

(6) $$\begin{array}{c} h_{t}=o_{t}*\;\tanh\left(c_{t}\right)\text{,} \end{array}$$

where *f*_*t*_, *i*_*t*_, and *o*_*t*_ denote the forget gate, input gate, and output gate, respectively, *h*_*t*−1_, *W*, and *b* are the output of the previous hidden layer state, weight, and bias of gate neurons, and *c*_*t*_′ and *c*_*t*_ are the cell state and the candidate of cell state.

LSTM is a proposed solution to overcome short-term memory problems by introducing internal gates mechanisms that regulate the flow of information. However, LSTM model only encodes information from front to back and cannot capture comprehensive semantic information. BiLSTM is actually two LSTMS that can better capture bidirectional semantics. One LSTM processes the sequence in the forward direction, and the other one processes the sequence in the reverse direction, and then, the output of the two LSTMS is combined. BiLSTM model is used to learn the dependencies between words in sentences and combine the sentence-level features in order to more fully express the sentence features. BiLSTM model is used to enhance feature of word vectors, and the state of the *t*-th position *h*_*t*_ is as follows:

(7) $$\begin{array}{c} h_{t}=\left[\overset{⟶}{h_{t}}⊕\overset{\leftarrow }{h_{t}}\right]\text{,} \end{array}$$

where $\overset{⟶}{h_{t}}$ and $\overset{\leftarrow }{h_{t}}$ are the forward hidden layer state and the backward hidden layer state, respectively.

### 3.4. Other and Linear Layers

After the feature process in BiLSTM, the processing word vectors *X*′ are generated. The dropout layer randomly sets input elements to zero with a given probability 50% to prevent overfitting. In final, *X*′ and *y* are connected and inputted to linear layer to generate emotion *F*, which is used to conduct emotion analysis of dialogue.

## 4. Experimental Results and Analysis

In this section, experiments are conducted to verify the efficiency of the proposed method on two real datasets. Our method is compared with the state-of-the-art emotion classification models.

### 4.1. Experiment Environment and Dataset

The experiments are executed on the server with an GPU RTX 3090@24 G bytes video RAM and AMD (EPYC 7543) 32-Core Processor. The server is running on Ubuntu 18.04 (64 bits) operating system, PyTorch 1.9.0 with GPU support only, and Python 3.8.

The first dataset is multimodal emotion lines dataset (MELD) [30] that includes text, audio, and video information. MELD contains more than 1,400 dialogues, totaling 13,000 utterances from the TV-series Friends. We choose text data as the first experiment dataset that contains seven emotions, namely anger, disgust, sadness, joy, neutral, surprise, and fear.

The second dataset used is high-quality multiturn dialogue dataset (DailyDialog) [31], which is only text data with low noise and reflects variety topics of daily life without fixed speaker. DailyDialog also contains seven emotions that are neutral, happiness, surprise, sadness, anger, disgust, and fear. 12,218 conversations with 103,607 sentences are in DailyDialog, which is the large data scale.

### 4.2. Evaluation Metrics

In order to evaluate the efficiency of the proposed method, as statistical measures for classification models, precision and recall are used in this paper. In general, the higher value of precision and recall, the better effect of classification model. The formula of the precision is as follows:

(8) $$\begin{array}{c} Precision=\frac{TP}{TP+FP}\text{,} \end{array}$$

where *TP* is the number of true positive samples, and *FP* is the number of false positive samples.

(9) $$\begin{array}{c} Recall=\frac{TP}{TP+FN}\text{,} \end{array}$$

where *FN* is the number of false negative samples.

However, precision and recall contradict each other sometimes. The most common method is *F*1-score that considers comprehensively and combines the results of precision and recall. The higher value of *F*1-score can show that the classification model is more effective. The formula of the *F*1-score is as follows:

(10) $$\begin{array}{c} F1-scroe=\frac{2*Recall*Precision}{Recall+Precision}\text{.} \end{array}$$

In this paper, we introduce weighted avg precision, recall, and *F*1-score that use the percentage of the number of samples from each classification in the total number of samples from all classification as the weight. In addition, FLOPs and params are the number of floating-point operations and the number of arguments that are required to all network models.

### 4.3. Baseline Methods

In order to validate the proposed model, some baselines are as follows.

  BERT-BiLSTM-CNN [27]: It combines the advantages of BERT embedding, BiLSTM, and TextCNN to capture local correlation and retain context information.
  BERT-BiGRU-CNN: This baseline replaces BiLSTM in BERT-BiLSTM-CNN with BiGRU, which has the simpler structure and calculation than BiLSTM.
  BERT-BiLSTM-attention: A classification model based on a BERT embedding and bidirectional LSTM which is combined with self-attention mechanism.
  BERT-(BiLSTM + CNN): This framework is based on BERT embedding and the double channel that is combined with BiLSTM and CNN to capture local correlation and retain context information.
  BERT-(BiLSTM-attention + CNN) [5]: It is an enhanced BERT-(BiLSTM + CNN) by adding self-attention mechanism to BiLSTM.
  BiBERT-BiLSTM: Our model is denoted by BiBERT-BiLSTM, which is based on BERT embeddings and BiLSTM. BERT extracts word-level and sentence-level vectors from the inputting data and is embed into the neural network architecture. And then word-level vectors are combined with BiLSTM to concatenate sentence-level vectors for emotion analysis.

### 4.4. Comparison with Baseline Methods

The first experiment results involved comparing all the different baseline methods on MELD dataset. The results are shown in Table 1.

**Figure 3:**
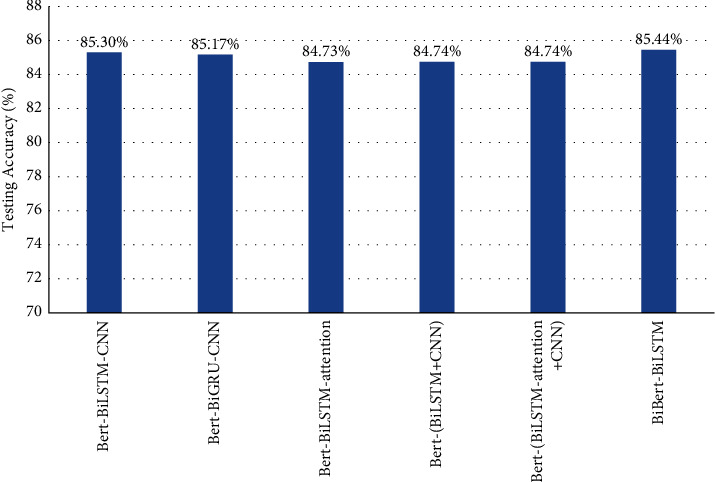
Testing accuracy of BiBERT-BiLSTM with baselines on DailyDialog.

From the results, BERT-BiLSTM-CNN and BERT-BiGRU-CNN have similar experiment results due to the similar model constructs. BERT-BiLSTM-attention outperforms BERT-BiLSTM-CNN and BERT-BiGRU-CNN. BERT-BiLSTM-attention has BiLSTM to learn the context of the dialogue and includes a self-attention mechanism so as to focus on the emotion classification features of dialogue. Compared to the single-channel methods, the double-channel methods also have better performance. BERT-(BiLSTM + CNN) and BERT-(BiLSTM-attention + CNN) use BERT embedding to extract word-level feature, which is input into BiLSTM and CNN for learning the context and local correlation, respectively. They achieve a performance close to BERT-BiLSTM-CNN and BERT-BiGRU-CNN. BERT-(BiLSTM-attention + CNN) has an advantage over BERT-(BiLSTM + CNN) by adding self-attention mechanism to BiLSTM. The proposed model BiBERT-BiLSTM uses BERT to extract word-level and sentence-level vectors, which have more comprehensive semantic features. BiBERT-BiLSTM outperforms all baselines. The experiment results of DailyDialog are in Figure 3. The testing accuracy of BiBERT-BiLSTM is 85.44% and the best, which outperforms BERT-BiLSTM-CNN by 0.14%, BERT-BiGRU-CNN by 0.27%, BERT-BiLSTM-attention by 0.71%, BERT-(BiLSTM + CNN) by 0.70%, and BERT-(BiLSTM-attention + CNN) by 0.70%.

We also evaluate the performance of all models.  Table 2 shows comparisons between BiBERT-BiLSTM and all baselines on model size (params) and complexity (giga floating-point operations). BiBERT-BiLSTM uses BERT to extract word-level and sentence-level vectors that have twice as much computation as other models with word-level vector only. At the same time, the params of all models are basically similar.

### 4.5. Ablation Study

To comprehensively test the validity of the proposed method, we conduct ablation study.  Table 3 shows comparisons between BiBERT-BiLSTM and the two main baselines on MELD dataset. BiBERT-BiLSTM outperforms BERT-BiLSTM and BERT-Sentence. The baseline BERT-BiLSTM only uses word-level vectors to BiLSTM for learning the context of dialogue and dismisses sentence-level vectors. BERT-Sentence only retains sentence-level vectors by BERT embedding and dismisses word-level vectors.

**Figure 4:**
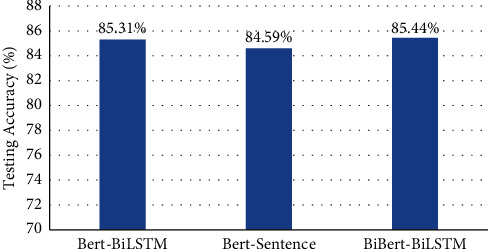
Testing accuracy of BiBERT-BiLSTM with ablation models on DailyDialog.

The experiment results of ablation study on DailyDialog are in Figure 4. The testing accuracy of BiBERT-BiLSTM is 85.44% and the best, which outperforms BERT-BiLSTM by 0.13% and BERT-Sentence by 0.85%.

### 4.6. Discussion

**Figure 5:**
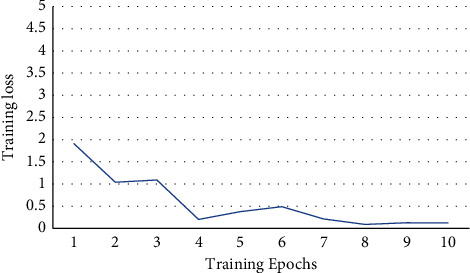
Training loss of BiBERT-BiLSTM on MELD.

In order to ensure the validity of the experiment, the various parameters on all models are in agreement in simulation environment. The same settings of parameters are based on the existed research: the batch size is 32, the number of iterations is 10, the hidden dimension is 384, respectively, the learning rate is 0.0002, and max length of sentence is 100. The training loss obtained during the experimentation is shown in Figure 5. From the simulation, the training loss stabilizes at about 0.2.

The experiment results of two real dialogue datasets prove the validity of the proposed method. BERT is a feature selection model that extracts word-level and sentence-level vectors from the inputting data. The proposed double-channel method has better performance by comparing with single-channel method. Then, word-level vectors are combined with BiLSTM that is used to enhance feature. The word-level vectors concatenate sentence-level vectors for emotion analysis. Experimental results show that BiBERT-BiLSTM is efficient.

## 5. Conclusions

Many researchers have conduct emotion analysis by different models and provide various methods for emotion classification. Based on the existed research, the architecture based on BERT embeddings and BiLSTM is proposed and constructed to determine emotion from dialogue. BERT extracts word-level and sentence-level vectors from the input data and is embed into the neural network architecture. And then, word-level vectors are combined with BiLSTM to concatenate sentence-level vectors for emotion analysis. To evaluate the proposed emotion classification model, experiments are conducted on two real dialogue datasets. The experimental results show that the proposed method significantly outperforms the baselines. In the future, we plan to extend sentence-level vectors for improving the accuracy of the model. In addition, we also plan to apply the emotion analysis model in dialogue system to generate emotional dialogue.

## Figures and Tables

**Table 1:** Comparison of the weighted average of all models.

Models	Precision	Recall	*F*1-score
BERT-BiLSTM-CNN	0.5900	0.5795	0.5804
BERT-BiGRU-CNN	0.5932	0.5864	0.5841
BERT-BiLSTM-attention	0.5975	0.5972	0.5895
BERT-(BiLSTM + CNN)	0.5902	0.5752	0.5766
BERT-(BiLSTM-attention + CNN)	0.5963	0.5818	0.5862
BiBERT-BiLSTM	0.6020	0.6011	0.5981

**Table 2:** Comparison of the performance of all models.

Models	GFLOPs	Model size (# params) (M)
BERT-BiLSTM-CNN	583.4	115.72
BERT-BiGRU-CNN	594.7	117.50
BERT-BiLSTM-attention	589	116.58
BERT-(BiLSTM + CNN)	594.7	117.50
BERT-(BiLSTM-attention + CNN)	594.7	117.50
BiBERT-BiLSTM	1132.6	116.58

**Table 3:** Results of ablation study experiment on the weighted avg.

Models	Precision	Recall	*F*1-score
BERT-BiLSTM	0.5886	0.5945	0.5872
BERT-sentence	0.5905	0.5938	0.5899
BiBERT-BiLSTM	0.6020	0.6011	0.5981

## Data Availability

The data used to support the findings of this study are available from the corresponding author upon request.

## References

[1] Huang J., Lee W. (2022). Exploring the effect of emotions in human–machine dialog: an approach toward integration of emotional and rational information. *Knowledge-Based Systems*.

[2] Jia K. (2022). Sentiment classification of microblog: a framework based on BERT and CNN with attention mechanism. *Computers & Electrical Engineering*.

[3] Mccallum A., Nigam K. A comparison of event models for Naive Bayes text classification.

[4] Cortes C., Vapnik V. (1995). Support-vector networks. *Machine Learning*.

[5] Gupta B., Prakasam P., Velmurugan T. (2022). Integrated BERT embeddings, BiLSTM-BiGRU and 1-D CNN model for binary sentiment classification analysis of movie reviews. *Multimedia Tools and Applications*.

[6] Deerwester S., Dumais S. T., Furnas G. W., Landauer T. K., Harshman R. (1990). Indexing by latent semantic analysis. *Journal of the American Society for Information Science*.

[7] Thomas H. Probabilistic latent semantic analysis.

[8] Blei D. M., Ng A. Y., Jordan M. I. (2003). Latent dirichlet allocation. *Journal of Machine Learning Research*.

[9] Wang J., Xu W., Yan W., Li C. Text similarity calculation method based on hybrid model of LDA and TF-IDF.

[10] Shao D., Li C., Huang C., Xiang Y., Yu Z. (2022). A news classification applied with new text representation based on the improved LDA. *Multimedia Tools and Applications*.

[11] Wu Z., Lei L., Li G. (2017). A topic modeling based approach to novel document automatic summarization. *Expert Systems with Applications*.

[12] Tomas M., Ilya S., Kai C., Greg C., Jeffrey D. Distributed representations of words and phrases and their compositionality.

[13] Devlin J., Chang M. W., Lee K. (2018). BERT: pre-training of deep bidirectional transformers for language understanding. https://arxiv.org/abs/1810.04805.

[14] Saga T., Tanaka H., Iwasaka H., Nakamura S. (2022). Multimodal prediction of social responsiveness score with BERT-based text features. *IEICE - Transactions on Info and Systems*.

[15] Zhang Z., Zhang Y., Li X., Qian Y., Zhang T. (2022). BMCSA: multi-feature spatial convolution semantic matching model based on BERT. *Journal of Intelligent and Fuzzy Systems*.

[16] Eke C. I., Norman A. A., Shuib L. (2021). Context-based feature technique for sarcasm identification in benchmark datasets using deep learning and BERT model. *IEEE Access*.

[17] Gao J. Chinese sentiment classification model based on pre-trained BERT.

[18] Chen X., Cong P., Lv S. (2022). A long-text classification method of Chinese news Based on BERT and CNN. *IEEE Access*.

[19] Palani S., Rajagopal P., Pancholi S. (2021). T-BERT-Model for sentiment analysis of micro-blogs integrating topic model and BERT. https://arxiv.org/abs/2106.01097.

[20] Wan C. X., Li B. (2022). Financial causal sentence recognition based on BERT-CNN text classification. *The Journal of Supercomputing*.

[21] Hochreiter S., Schmidhuber J. (1997). Long short-term memory. *Neural Computation*.

[22] Song H., Li G., Hou S., Qu Y., Liang H., Bai X. Translate and summarize complaints of patient to electronic health record by BiLSTM-CNN attention model.

[23] Ertugrul A. M., Karagoz P. Movie genre classification from plot summaries using bidirectional LSTM.

[24] Liu Z., Kang X., Ren F. (2022). Improving speech emotion recognition by fusing pre-trained and acoustic features using transformer and BiLSTM. *Intelligent Information Processing XI*.

[25] Ultes S. (2020). Improving interaction quality estimation with BiLSTMs and the impact on dialogue policy learning. https://arxiv.org/abs/2001.07615.

[26] Gong L., Zhang X., Chen T., Zhang L. (2021). Recognition of disease genetic information from unstructured text data based on BiLSTM-CRF for molecular mechanisms. *Security and Communication Networks*.

[27] Jiang X., Song C., Xu Y., Li Y., Peng Y. (2022). Research on sentiment classification for netizens based on the BERT-BiLSTM-TextCNN model. *PeerJ Computer Science*.

[28] Enamoto L., Santos A. R., Maia R., Weigang L., Filho G. P. R. (2022). Multi-label legal text classification with BiLSTM and attention. *International Journal of Computer Applications in Technology*.

[29] Deng J., Cheng L., Wang Z. (2021). Attention-based BiLSTM fused CNN with gating mechanism model for Chinese long text classification. *Computer Speech & Language*.

[30] Poria S., Hazarika D., Majumder N., Naik G., Cambria E., Mihalcea R. MELD: a multimodal multi-party dataset for emotion recognition in conversations.

[31] Li Y., Su H., Shen X., Li W., Cao Z., Niu S. DailyDialog: a manually labelled multi-turn dialogue dataset.

